# Efficacy of Individual Bacteriophages Does Not Predict Efficacy of Bacteriophage Cocktails for Control of *Escherichia coli* O157

**DOI:** 10.3389/fmicb.2021.616712

**Published:** 2021-02-24

**Authors:** Yan D. Niu, Hui Liu, Hechao Du, Ruiqiang Meng, El Sayed Mahmoud, Guihua Wang, Tim A. McAllister, Kim Stanford

**Affiliations:** ^1^Department of Ecosystem and Public Health, Faculty of Veterinary Medicine, University of Calgary, Calgary, AB, Canada; ^2^Hohhot Bureau of Ecology and Environment, Hohhot, China; ^3^Lethbridge Research and Development Centre, Agriculture and Agri-Food Canada, Lethbridge, AB, Canada; ^4^College of Animal Science and Technology, Jinling Institute of Technology, Nanjing, China; ^5^Inner Mongolia C. P. Livestock Husbandry Co., Ltd., Hohhot, China; ^6^School of Applied Computing, Faculty of Applied Science and Technology, Sheridan College, Oakville, ON, Canada; ^7^College of Veterinary Medicine, Shandong Agricultural University, Tai’an, China; ^8^Department of Biological Science, University of Lethbridge, Lethbridge, AB, Canada

**Keywords:** bacteriophages (phages), *Escherichia coli* O157, phage biocontrol, phage cocktail, phage-phage interactions

## Abstract

Effectiveness of bacteriophages AKFV33 (*Tequintavirus*, T5) and AHP24 (*Rogunavirus*, T1), wV7 (*Tequatrovirus*, T4), and AHP24S (*Vequintavirus*, rV5), as well as 11 cocktails of combinations of the four phages, were evaluated *in vitro* for biocontrol of six common phage types of *Escherichia coli* O157 (human and bovine origins) at different multiplicities of infection (MOIs; 0.01–1,000), temperatures (37 or 22°C), and exposure times (10–22 h). Phage efficacy against O157 was highest at MOI 1,000 (*P* < 0.001) and after 14-18 h of exposure at 22°C (*P* < 0.001). The activity of individual phages against O157 did not predict the activity of a cocktail of these phages even at the same temperature and MOI. Combinations of phages were neutral (no better or worse than the most effective constituent phages acting alone), displayed facilitation (greater efficacy than the most effective constituent phages acting alone), or antagonistic (lower efficacy than the most effective constituent phages acting alone). Across MOIs, temperatures, exposure time, and O157 strains, a cocktail of T1, T4, and rV5 was most effective (*P* < 0.05) against O157, although T1 and rV5 were less effective (*P* < 0.001) than other individual phages. T5 was the most effective individual phages (*P* < 0.05), but was antagonistic to other phages, particularly rV5 and T4 + rV5. Interactions among phages were influenced by phage genera and phage combination, O157 strains, MOIs, incubation temperatures, and times. Based on this study, future development of phage cocktails should, as a minimum, include confirmation of a lack of antagonism among constituent phages and preferably confirmation of facilitation or synergistic effects.

## Importance

A combination of multiple phages is generally recommended to thwart resistance development by bacteria, but treatment with multiple phages has not always resulted in superior efficacy when compared with treatment with a single phage. This study is the first systematically evaluating interactions among multiple phages resulting in neutral, improved, or reduced efficacy of *Escherichia coli* O157 biocontrol. Selecting phages for inclusion in cocktails solely based on great individual efficacy may not ensure maximal cocktail efficacy. For example, the most effective cocktail across MOIs, temperatures, O157 strains, and exposure times was a three-phage combination, which includes two phages with the lowest individual efficacy. Temperature and duration of phage exposure also must be considered, as a cocktail highly effective at 22°C was one of the least effective at 37°C. Therefore, carefully screening phage combinations against targeted bacteria to ensure phage facilitation or synergy or, at minimum, a lack of phage antagonism under required environmental conditions is crucial to achieving enhanced biocontrol and therapeutic outcomes.

## Introduction

Bacteriophages (phages) are natural predators of bacteria, targeting specific bacterial species or strains. Owing to their ability to self-replicate, high specificity against target bacteria, and ubiquity within the environment (Abedon, 2009; Nobrega et al., 2015), phages have been recognized as a potential alternative to antimicrobials to treat bacterial infections (Kortright et al., 2019). In March 2014, the United States National Institute of Allergy and Infectious Diseases listed phage therapy as one of seven prongs in its plan to combat antimicrobial resistance (Reardon, 2014). Numerous phages have shown their ability to control bacterial infections in humans (Kortright et al., 2019), livestock (Carvalho et al., 2017), aquaculture (Culot et al., 2019), and plants (Buttimer et al., 2017), as well as to decontaminate processed foods and agricultural products (Moye et al., 2018). To date, a variety of chronic and acute bacterial infections of the skin, respiratory tract, and gastrointestinal systems, representing over 20 different species of pathogens, have been controlled in humans using phage therapy (Cisek et al., 2017; Kortright et al., 2019). Nevertheless, failures of phage treatment are common, with the development of bacterial resistance to phages being one of the causative factors (Chan et al., 2013). As a result, the inclusion of multiple phages in cocktails is commonly recommended as a means of avoiding resistance (Brüssow, 2012; Chan et al., 2013), as it is theorized that resistance is less likely to develop against a cocktail than a single phage. However, *in vivo* studies have demonstrated that phage cocktails do not always offer superior efficacy as compared with the administration of a single phages. For instance, Watanabe et al. (2007) reported that treatment with a cocktail of phages with activity against *Pseudomonas aeruginosa* in mice was no more effective than a single phage. In addition, Bourdin et al. (2014) found that a cocktail of three T4-like phages exhibited a narrower host range against *Escherichia coli* strains in broth culture as compared with when these phages were used individually. These previous studies indicate that combining phages into a cocktail may create phage antagonism, making the actual lytic activity less than the sum of its constituents.

*Escherichia coli* O157 is a worldwide health threat (Keithlin et al., 2014; Majowicz et al., 2014), causing approximately 63,000 illnesses, 2,000 hospitalizations, and 20 deaths annually in the United States (Scallan et al., 2011). Ruminants are major reservoirs of *E. coli* O157, which colonizes the gastrointestinal tract and is passed to the environment through feces (Grauke et al., 2002). Lytic phages have been shown to reduce populations of Shiga toxin-producing *E. coli* O157:H7 *in vitro* (Niu et al., 2009a; Sabouri et al., 2017) and within food matrices (Liu et al., 2015; Vikram et al., 2020). In previous studies, we have isolated various phages from cattle and their environment (Niu et al., 2009b; Hallewell et al., 2014). Several of these including vB_EcoS_AKFV33 of *Tequintavirus* (T5) (Niu et al., 2012, 2020), vB_EcoS_AHP24 of *Rogunavirus* (T1) (Niu et al., 2014), V7 of *Tequatrovirus* (T4) (Niu et al., 2009a; Kropinski et al., 2012), and vB_EcoM_AHP24S of *Vequintavirus* (rV5) (Niu et al., 2015) were shown to have a broad host range and strong lytic activity against *E. coli* O157. Therefore, the objectives of the study were to use *E. coli* O157-infecting phages T5, T1, T4, and rV5 as models to (1) compare the effectiveness of single phages vs. phage cocktails in all possible combinations *in vitro*; (2) compare phage efficacy as impacted by ambient temperature, exposure time, MOI, and bacterial strain; and (3) identify potential interactions among phages.

## Materials and Methods

### Bacteriophages, Bacteria, and Media

*Escherichia coli* O157-infecting phages T5, T1, rV5, and T4 were cultured individually as described by Liu et al. (2015) and enumerated by soft agar overlay plaque assay (Sambrook and Russell, 2001) using *E. coli* O157:H7 strain R508 (Phage type 14) as a host. Each phage was incubated with the host at 37°C for 4-6 h until complete lysis occurred. The lysed culture was then centrifuged and filtered through a 0.2-μm SFCA serum filter (Nalgene, Rochester, NY, United States). Stock preparations of T5, T1, T4, and rV5 contained 3.68 × 10^10^, 2.74 × 10^9^, 8.75 × 10^8^, and 9.78 × 10^9^ PFU ml^–1^, respectively, and were maintained at 4°C for up to 3 months. To achieve a final titer of ∼10^8^ PFU/ml for lysis kinetics, individual phage preparations were diluted using modified tryptic soy broth (mTSB) containing 10 mmol l^–1^ of magnesium sulfate, whereas 11 phage cocktails were prepared by mixing equal volumes of each filtered phage stock in all possible combinations (Table 1).

**TABLE 1:** Odds ratios comparing the likelihood of superior phage efficacy against *E. coli* O157 at different incubation temperatures, incubation times, MOIs, phages, and strains of *E. coli* O157.

Factors^§^	Percentage (%) of optical turbidity measurement <0.01	Odds ratio	95% CI^#^	*P*-value*
**Temperature, time**				
22°C, 16 h	75^a^	2.96	2.58-3.40	<0.001
22°C, 18 h	75^a^	2.88	2.51-3.31	<0.001
22°C, 14 h	75^a^	2.80	2.44-3.21	<0.001
37°C, 8 h	72^b^	2.40	2.10-2.76	<0.001
22°C, 12 h	72^bc^	2.32	2.02-2.66	<0.001
22°C, 10 h	71^bcd^	2.13	1.86-2.43	<0.001
22°C, 20 h	71^cd^	2.04	1.79-2.34	<0.001
37°C, 6 h	70^d^	1.87	1.63-2.14	<0.001
37°C, 10 h	67^e^	1.55	1.36-1.77	<0.001
22°C, 22 h	61^h^	Referent		
**MOIs**				
1,000	88^a^	23.39	20.62-26.52	<0.001
100	81^b^	11.57	10.35-12.94	<0.001
10	79^c^	9.20	8.26-10.25	<0.001
1	73^d^	5.97	5.39-6.64	<0.001
0.1	61^e^	2.65	2.41-2.92	<0.001
0.01	44^f^	Referent		
**Phages**				
T1 + T4 + rV5	89^a^	109.12	88.88-133.98	<0.001
T5 + T1 + T4 + rV5	87^ab^	90.57	74.21-110.52	<0.001
T5 + T1 + T4	86^b^	83.03	68.21-101.07	<0.001
T5 + T1	86^b^	81.64	67.10-99.32	<0.001
T5 + T1 + rV5	83^c^	60.30	49.95-72.78	<0.001
T1 + T4	82^c^	56.37	46.78-67.94	<0.001
T5	81^c^	51.61	42.91-62.07	<0.001
T5 + T4	79^d^	41.66	34.78-49.89	<0.001
T5 + T4 + rV5	67^e^	17.74	14.99-20.99	<0.001
T4 + rV5	65^ef^	15.95	13.49-18.85	<0.001
T5 + rV5	64^ef^	15.45	13.08-18.25	<0.001
T4	63^f^	14.41	12.21-17.02	<0.001
T1 + rV5	59^g^	11.34	9.62-13.37	<0.001
T1	52^h^	7.44	6.32-8.74	<0.001
rV5	19^i^	Referent		
**Bacteria**				
*E. coli* O157:H7 CO281-31N (H, PT8)	88^a^	27.95	24.15-32.34	<0.001
*E. coli* O157:H7 H4420N (B, PT87)	80^b^	11.42	10.06-12.96	<0.001
*E. coli* O157:H7 EDL933 (H, PT21)	78^c^	9.88	8.73-11.19	<0.001
*E. coli* O157:H7 E318N (H, PT4)	74^d^	7.06	6.26-7.96	<0.001
*E. coli* O157:H7 R508N (B, PT14)	73^d^	6.45	5.72-7.26	<0.001
*E. coli* O157:NM E32511 (H, PT31)	71^e^	5.60	4.98-6.30	<0.001
All 7 mixture	61^f^	2.97	2.66-3.32	<0.001
*E. coli* O157:H7 3081 (B, PT43)	43^g^	Referent		

^§^H, Human; B, Bovine; PT, phage type. ^#^CI, confidence interval for the odds ratio. *P-value for the likelihood of superior phage efficacy as affected by different incubation temperatures, incubation times, MOIs, phages, and strains of *E. coli* O157. ^*a–i*^Within same factors, means different letters differs (*P* < 0.05).

Standard laboratory strains of *E. coli* O157 representing the seven most common phage types (PTs 4, 8, 14, 21, 31, 43, and 87) isolated from cattle and humans were used to evaluate phage efficacy (Table 1). To prepare *E. coli* O157 inoculum, each strain was cultured in 10 ml of TSB at 37°C for 18 h to reach 9 log_10_ CFU/ml. Mixtures of the seven strains were prepared by mixing an equal volume of an overnight culture of each strain. Overnight cultures of each *E. coli* O157 strain or a mixture of all seven strains were serially diluted using mTSB containing 10 mmol l^–1^ of magnesium sulfate to achieve 4–5 log_10_ CFU/ml.

### Phage *in vitro* Lysis Kinetics

Each individual phages or each phage cocktail at the titer of ∼10^8^ PFU/ml (20 μl) was serially diluted 10-fold in 180 μl of mTSB in 96-well flat-bottom microplates (Nunc, Fisher, AB, Canada). Duplicate wells of each phage dilution were then inoculated with 20 μl of diluted overnight cultures of individual *E. coli* O157 strains or the seven-strain mixture to achieve an MOI ranging from 0.01 to 1,000 (Table 1). Negative control wells were prepared in each microplate that contained mTSB inoculated with bacteria only and wells with mTSB only served as a blank. The plates were incubated at 37°C for 10 h or at 22°C for 22 h. At 2-h intervals, plates were removed from the incubator and mixed at 720 rpm for 30 s and examined for optical density at 600 nm (OD_600_) using a Synergy^TM^ HT multimode microplate reader (BioTek, Winooski, VT, United States). Blank values were subtracted from absorbance measures at 600 nm to give a final corrected optical density.

### Statistical Analysis

Results from phage lysis kinetics were compiled from two independent experiments. Superior phage efficacy was defined as complete inhibition of bacterial growth (i.e., OD_600_ ≤ 0.01). Influence of incubation temperature, time, MOIs, *E. coli* O157 strains, and phage types on phage efficacy were analyzed using GLIMMIX with random measures. Odds ratios were calculated to compare the likelihood of complete inhibition (OD_600_ ≤ 0.01) of bacterial growth for varying incubation temperatures, MOIs, *E. coli* O157 strains, and phages with cohorts incubated at 22°C for 22 h, at an MOI = 0.01, using phage rV5 and *E. coli* O157 strain 3081 used as the referents. Referents would be the temperature, MOI, or *E. coli* O157 strain with the least likelihood of producing complete inhibition of bacterial growth. A phage cocktail with an odds ratio of 100 would then be 100 times more likely to completely inhibit bacterial growth compared with the referent. For each bacteria and temperature, the OD_600_ values were square-root transformed and then analyzed using the MIXED model, and least-squares were used to differentiate means (*P* < 0.05). Panels A-H were assigned to each phage treatment, of which overall anti-O157 efficacy across time and MOIs statistically differed (*P* < 0.05) within each strain (Table 2).

**TABLE 2:** Overall phage efficacy against *E. coli* O157 at 37°C.

3081				CO281-31N				E318N				E32511			
Panel^§^	Phage treatment	Mean OD	Best MOIs*	Panel	Phage treatment	Mean OD	Best MOIs	Panel	Phage treatment	Mean OD	Best MOIs	Panel	Phage treatment	Mean OD	Best MOIs
A	T4	0.018	>0.01	A	T1 + T4 + rV5	0	Any	A	T1 + T4 + rV5	0	Any	A	T1 + T4 + rV5	0	Any
	T5 + T4	0.025	>0.01		T5 + T4	0	Any		T5	0.005	Any		T5 + T4 + rV5	0.002	Any
B	T1 + T4	0.041	>0.01		T5 + T1 + T4	0	Any		T5 + T1	0.006	Any		T4 + rV5	0.002	Any
	T5 + T1 + T4	0.055	>0.1		T5 + T1 + rV5	0	Any	B	T5 + rV5	0.008	≠100		T5	0.003	Any
	T1 + T4 + rV5	0.069	>0.1		T1 + T4	0	Any		T5 + T4	0.009	>0.1		T5 + rV5	0.004	Any
	T5 + T1 + T4 + rV5	0.070	>0.1		T5 + rV5	0	Any		T5 + T1 + T4 + rV5	0.015	>1	B	T5 + T1 + rV5	0.005	>0.01
	T5 + T4 + rV5	0.075	>0.1		T5 + T4 + rV5	0	Any		T4	0.015	>10		T5 + T1	0.006	>0.01
C	T4 + rV5	0.085	>0.1		T4 + rV5	0	Any		T5 + T1 + rV5	0.021	0.1, 1		T4	0.007	>0.01
D	T5	0.371	1,000	B	T5	0.004	Any	C	T1 + T4	0.023	>10		T5 + T4	0.008	>0.1
	T5 + T1	0.390	1,000		T5 + T1	0.005	Any		T5 + T4 + rV5	0.025	>0.1		T5 + T1 + T4 + rV5	0.011	>0.1
E	T5 + T1 + rV5	0.421	1,000		T4	0.005	Any		T4 + rV5	0.026	>0.1		T1 + T4	0.012	>0.1
	T5 + rV5	0.424	1,000		rV5	0.005	Any		T1 + rV5	0.033	1,000		T5 + T1 + T4	0.012	0.1,1,10
F	T1	0.479	NA		T5 + T1 + T4 + rV5	0.006	Any	D	T5 + T1 + T4	0.076	>1	C	rV5	0.098	100
	rV5	0.506	NA	C	T1 + rV5	0.01	>0.01		T1	0.08	NA	D	T1 + rV5	0.106	>1
	T1 + rV5	0.515	NA	D	T1	0.336	NA		rV5	0.086	NA	E	T1	0.375	NA
**Average Mean OD**		0.236		**Average Mean OD**		0.025		**Average Mean OD**		0.029		**Average Mean OD**		0.043	

**Significance between panels**				**Significance between panels**				**Significance between panels**				**Significance between panels**			

A vs. B *P* < 0.01				A vs. B *P* < 0.001				A vs. B *P* < 0.01				A vs. B *P* < 0.01			
B vs. C *P* < 0.01				B vs. C *P* < 0.05				B vs. C *P* < 0.01				B vs. C *P* < 0.001			
C vs. D *P* < 0.001				C vs. D *P* < 0.001				C vs. D *P* < 0.01				C vs. D *P* < 0.01			
D vs. E *P* < 0.001												D vs. E *P* < 0.001			
E vs. F *P* < 0.001 [F: did not work as compared to control (*P* > 0.1)]															

**EDL933**				**H4420N**				**R508N**				**All 7 mixture**			
**Panel^§^**	**Phage treatment**	**Mean OD**	Best MOIs*	**Panel**	**Phage treatment**	**Mean OD**	**Best MOIs**	**Panel**	**Phage treatment**	**Mean OD**	**Best MOIs**	**Panel**	**Phage treatment**	**Mean OD**	**Best MOIs**

A	T1 + T4 + rV5	0	Any	A	T1 + T4 + rV5	0	Any	A	T1 + T4 + rV5	0	Any	A	T1 + T4 + rV5	0	Any
	T5 + T1	0.004	Any		T5	0	Any		T4 + rV5	0	Any	B	T5 + T1	0.007	>0.01
	T4	0.004	Any		T5 + T1	0	Any		T5 + T4 + rV5	0	Any		T5	0.007	>0.01
	T5 + rV5	0.004	Any	B	T5 + rV5	0	>0.01		rV5	0.002	Any		T5 + rV5	0.008	>0.01
	T5 + T1 + rV5	0.005	Any		T5 + T1 + T4 + rV5	0.000	>0.01		T5	0.002	Any		T5 + T1 + rV5	0.008	>0.01
	T1 + T4	0.005	Any		T5 + T1 + T4	0.000	>0.01	B	T4	0.005	>0.01		T1 + T4	0.014	>0.1
	T5	0.006	Any		T5 + T1 + rV5	0.001	>0.01		T5 + T1 + rV5	0.005	>0.01		T4 + rV5	0.016	>0.1
	T5 + T4	0.006	Any	C	T5 + T4	0.013	>0.1		T5 + T1	0.005	>0.01		T5 + T1 + T4	0.02	>0.1
	T5 + T1 + T4 + rV5	0.006	Any		T5 + T4 + rV5	0.021	>0.1		T1 + rV5	0.005	>0.01		T5 + T4 + rV5	0.021	>0.01
B	T5 + T1 + T4	0.014	≠10		T4	0.027	NA		T5 + rV5	0.006	≠1	C	T4	0.025	1,000
C	T5 + T4 + rV5	0.053	>0.01		T1 + T4	0.027	>0.01		T1 + T4	0.006	>0.1		T5 + T4	0.026	10, 1,000
	T4 + rV5	0.058	>0.01		T4 + rV5	0.035	>0.1		T5 + T1 + T4	0.008	>0.1		T5 + T1 + T4 + rV5	0.038	>0.01
D	T1	0.232	NA	D	T1 + rV5	0.052	NA	C	T5 + T4	0.026	1,000	D	rV5	0.143	NA
	T1 + rV5	0.24	NA	E	T1	0.063	1,000	D	T1	0.042	NA		T1	0.149	NA
E	rV5	0.417	NA		rV5	0.075	NA		T5 + T1 + T4 + rV5	0.077	≠1 and 0.01		T1 + rV5	0.194	NA
**Average Mean OD**		0.070		**Average Mean OD**		0.021		**Average Mean OD**		0.013		**Average Mean OD**		0.045	

**Significance between Panels**				**Significance between Panels**				**Significance between Panels**				**Significance between Panels**			
A vs. B *P* < 0.05				A vs. B *P* < 0.05				A vs. B *P* < 0.05				A vs. B *P* < 0.001			
B vs. C *P* < 0.001				B vs. C *P* < 0.05				B vs. C *P* < 0.001				B vs. C *P* < 0.001			
C vs. D *P* < 0.001				C vs. D *P* < 0.001				C vs. D *P* < 0.001				C vs. D *P* < 0.001			
D vs. E *P* < 0.001				D vs. E *P* < 0.01											

^§^Panels A-F were assigned to each phage treatment of which overall anti-O157 efficacy across times and MOIs statistically differed (*P* < 0.05) within each strain. *Best MOIs represented complete inhibition of bacterial growth (OD600 ≤ 0.01) at each sampling time; NA, Not applicable – bacterial growth was not completely inhibited at any MOI. Symbol >indicates all concentrations greater than that listed, symbol ≠ indicates all other MOIs were superior to that listed.

Interactions among phages resulted in three outcomes (1) neutrality (N), where combining phages in a cocktail did not improve or reduce (*P* > 0.05) lytic efficacy as compared with the greatest efficacy demonstrated by individual constituent phages acting alone; (2) antagonism (A), where the cocktail reduced lytic efficacy (*P* < 0.05) as compared with the greatest efficacy demonstrated by individual constituent phages acting alone; and (3) facilitation (F), where a cocktail improved the lytic efficacy (*P* < 0.05) as compared with any of the constituent phages acting alone (Chaudhry et al., 2017). Using these criteria, N or A or F were assigned to each phage–bacterial strain combination at each sampling time, temperature, and MOI. The transformed interaction data were then analyzed using the GLIMMIX procedure with random measures. Odds ratios were calculated for the percentage of N or A or F with cohorts incubated at 22°C for 10 h, at an MOI = 1,000, using phage T5 + T1 and *E. coli* O157 strain CO281-31N as the referents. All analyses were conducted with SAS (SAS Institute, 1999).

## Results

**FIGURE 1:**
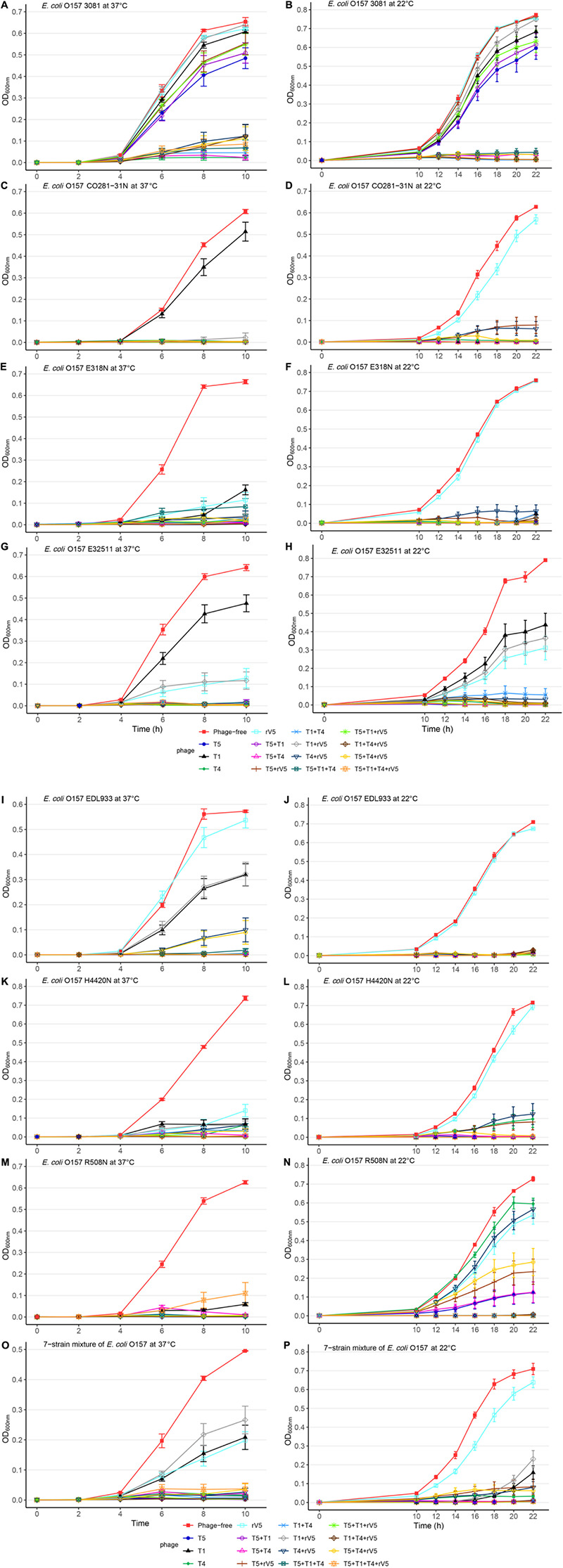
Growth curves of selected *E. coli* O157 strains at 22 and 37°C in individual and mixed cultures treated and not treated with phages across MOIs. **(A)** *E. coli* O157 3081 at 37°C; **(B)** *E. coli* O157 3081 at 22°C; **(C)** *E. coli* O157 C0281-31N at 37°C; **(D)** *E. coli* O157 C0281-31N at 22°C; **(E)** *E. coli* O157 E318N at 37°C; **(F)** *E. coli* O157 E318N at 22°C; **(G)** *E. coli* O157 E32511 at 37°C; **(H)** *E. coli* O157 E32511 at 22°C; **(I)** *E. coli* O157 EDL933 at 37°C; **(J)** *E. coli* O157 EDL933 at 22°C; **(K)** *E. coli* O157 H4420N at 37°C; **(L)** *E. coli* O157 H4420N at 22°C; **(M)** *E. coli* O157 R508N at 37°C; **(N)** at *E. coli* O157 R508N at 22°C; **(O)** seven-strain mixture of *E. coli* O157 at 37°C; **(P)** seven-strain mixture of *E. coli* O157 at 22°C. Bars present standard error of mean.

Generally, phage efficacy depended on incubation temperature (*P* < 0.001), time (*P* < 0.001), and MOI (*P* < 0.001) (Table 1). Overall, superior efficacy occurred after 14, 16, and 18 h of incubation at 22°C (*P* < 0.001) and at an MOI of 1,000 (*P* < 0.001). Across MOIs, phages, and O157 strains, anti-O157 efficacy of phages was 2.8 times [95% confidence interval (CI) 2.44-3.40, *P* < 0.001] more likely achieved between 14 and 18 h of incubation than at 22 h at 22°C. Across incubation temperatures, times, phages, and O157 strains, the likelihood of achieving superior efficacy at an MOI of 1,000 was >23 times (95% CI 20.62–26.52, *P* < 0.001) higher than at an MOI of 0.01. Across incubation temperatures, times, MOIs and O157 strains, the efficacy of individual phages did not predict the performance of cocktails (Table 1 and Figure 1). Phage T5 had markedly superior efficacy (*P* < 0.001) as compared with other individual phages. However, a combination of T5 and T4 resulted in a cocktail that was less effective against *E. coli* O157 than T5 alone (*P* < 0.05). Conversely, rV5 was the least effective (*P* < 0.001) of individual phages, but was a member of the most effective (*P* < 0.05) cocktail when combined with T1 and T4. As compared with rV5 alone, cocktails of T1 + T4 + rV5 and T5 + T1 + T4 + rV5 were 90–109 times (95% CI 74.12–133.98, *P* < 0.001) more likely to achieve superior efficacy. Adding T5 and preparing a four-phage cocktail did not further increase the lysis of *E. coli* O157 over that of the original three-phage cocktail (*P* = 0.09; Table 1).

Across incubation temperatures, times, MOIs, and phage tested, strains of *E. coli* O157 differed (*P* < 0.001) in their sensitivity to phages (Table 1). Strain CO281-31N was most sensitive (*P* < 0.001) to phages, of which growth was >27 times (95% CI 24.15–32.34, *P* < 0.001) more likely to be completely inhibited than strain 3081. Sensitivity of strains to phages decreased by an order of H4420N (*P* < 0.05), EDL933 (*P* < 0.001), E318N = R508N (*P* < 0.05), and E32511 (*P* < 0.001), a mixture of all *E. coli* strains (*P* < 0.001) and 3081 (*P* < 0.05). Phage effectiveness against each strain varied with temperatures, with MOIs of some individual phages and cocktails completely inhibiting bacterial growth at each sampling time. Across MOIs, anti-O157 efficacy of individual phages and cocktails were compared over 10 h of incubation at 37°C and 22 h of incubation at 22°C (Figure 1).

### Impact of Bacterial Strain on Phage Efficacy

#### CO281-31N

Among the strains assessed, CO281-31N was sensitive (*P* < 0.001) to all of the individual phages but T1 (Table 2 and Figure 1C). Irrespective of MOIs and incubation time, complete inhibition of bacterial growth (mean OD_600_ value = 0.002, *P* < 0.001) occurred with 13 of 15 individual phages or cocktails evaluated (panels A and B, Table 2 and Figure 1C). Moreover, with the exception of cocktails of T5 + T1, T5 + T1 + T4 + rV5 from panel B, and T1 + rV5 from panel C, phage cocktails were more effective (*P* < 0.001) at killing this strain than any single phages.

#### 3081

In contrast to CO281-31N, 3081 was the least sensitive to phages (mean OD_600_ value = 0.236, *P* < 0.001; Tables 1, 2). Comparing phage treatments, T4 alone exhibited the greatest efficacy (*P* < 0.01), which declined (*P* < 0.01), as other phages were added to generate a cocktail (panel B), except T5. As an individual phage, T5 (panel D) was able to completely lyse 3081 at each sampling time, although only at an MOI of 1,000, whereas 3081 was completely resistant to T1, rV5, and a cocktail of T1 + rV5 (panel E, *P* > 0.1; Figure 1A). Phage cocktails containing T4 (panels A and B) were able to completely inhibit bacterial growth at an MOI as low as 1 at each sampling time (Table 2 and Figure 1A).

#### E318N

E318N was highly sensitive (*P* < 0.001) to all individual phages and cocktails with a mean OD_600_ value of 0.025 (Table 2 and Figure 1E). Among treatments, T5 and cocktails of T1 + T4 + rV5 and T5 + T1 (panel A) were most effective (*P* < 0.001), completely inhibiting the bacterial growth at all MOIs and at each sampling time (Figure 1E). However, T1 and rV5 individually (panel D) were not capable of completely lysing this strain after 4 h at any of the MOIs evaluated (Figure 1E).

#### E32511

Similar to CO281-31N, E32511 was less sensitive (*P* < 0.001) to T1 than other phages (Table 2 and Figure 1G). T5 alone and cocktails of T1 + T4 + rV5, T5 + T4 + rV5, T4 + rV5, and T5 + rV5 (panel A) were more effective (*P* < 0.01) than other phage treatments, completely inhibiting bacterial growth at each MOI and sampling time (Table 2 and Figure 1G).

#### EDL933

This strain was the second-most susceptible to phages after CO281-31N, in that its growth was completely inhibited by T4 and T5 and seven cocktails containing T4 and/or T5 from panel A at all MOI (mean OD_600_ = 0.004, *P* < 0.05; Table 2). However, susceptibility to phages by EDL933 was not universal, as it was resistant to T1 and rV5 alone and in combination (Table 2 and Figure 1I).

#### H4420N

Similar to 318N, H4420N was sensitive to all the phages tested (average OD_600_ value = 0.021 across 15 phage treatments) with T1 + T4 + rV5, T5, and T5 + T1 being most effective (*P* < 0.05; Table 2). Unlike other bacterial strains, resistance to T4 was apparent after 8 h of incubation, even at the highest MOI, as bacterial growth was not completely inhibited (Figure 1K and Supplementary Figure 1). Another striking feature shown by this bacterium was that OD_600_ values with cocktails of T5 + rV5, T5 + T1 + T4, and T5 + T1 + rV5 at the two lowest MOIs increased slightly at 6 h but decreased to 0 after 8–10 h (Supplementary Figure 1).

#### R508N

Similar to E318N and H4420N, R508N was sensitive to each individual phage and cocktail evaluated, displaying a mean OD_600_ value of 0.013, only slightly above the cutoff for complete inhibition of bacterial growth (Table 2). This strain was highly susceptible to phage rV5, in contrast to other *E. coli* O157, which were generally most resistant to this phage (Table 2 and Figure 1M). Although this strain was sensitive to all individual phages and cocktails, the four-phage cocktail had lower lytic activity than all other phage treatments (*P* < 0.05), with the exception of T1.

### Mixture of the Seven Strains of *E. coli* O157

Similar to the results with individual *E. coli* O157 strains (excluding 3081), T1 + T4 + rV5 showed the greatest activity (*P* < 0.001) compared with individual phages and cocktails against the seven-strain bacterial mixture, completely inhibiting bacterial growth at each MOI and sampling time (Table 2 and Figure 1O). Of the individual phages, T5 was the most lytic (*P* < 0.001), and was able to completely inhibit bacterial growth at all but the lowest MOI. In contrast, T4 required an MOI of 1,000 to completely inhibit bacterial growth, and T1 and rV5 were not able to completely inhibit bacterial growth at any of the MOI evaluated (Figure 1O). T5 and some cocktails including T5 + T1, T5 + rV5, T5 + T1 + rV5, and T5 + T4 + rV5 exhibited the second highest efficacy (*P* < 0.001).

### Impact of Temperature on Phage Efficacy

Two of the phages (T5 and T4) did not differ in efficacy at 37 and 22°C (Tables 2, 3, *P* > 0.1). In contrast, the activity of both T1 and rV5 was temperature-dependent, with the former being more active at 22°C and the latter being more active at 37°C (*P* < 0.001). The improved lytic ability of T1 against CO281-31N (Figure 1D), H4420N (Figure 1L), and R508N (Figure 1N) was particularly evident at 22°C, as strains were completely inhibited at each MOI. At 37°C, phage T1 was not able to completely lyse CO281-31N or R508N at any MOI and only completely lysed H4420N at an MOI of 1,000 (Tables 2, 3 and Figures 1C,M,K and Supplementary Figure 1). At 22°C, adding rV5 to T4 and/or T5 reduced lysis (*P* < 0.05) of all bacterial strains as well as the seven-strain mixture, although in the case of R508N, T4 + rV5 had a higher OD_600_ (*P* < 0.05) than rV5 alone. At 37°C, T1 + T4 + rV5 was superior at lysing the seven-strain mixture and individual bacterial strains, except 3081. However, at 22°C, T1 + T4 + rV5 was not among the most effective preparations against EDL933, although this cocktail improved the lysis of strain 3081 as compared with that at 37°C. In addition, for the seven-strain mixture and strains CO281-31N, E318N, and E32511, no phage cocktail exhibited superior lytic efficacy as compared with T5 alone. Interestingly, at 22°C, T5 appeared to have less of a negative influence on the lytic ability of cocktails than at 37°C.

**TABLE 3:** Overall phage efficacy against *E. coli* O157 at 22°C.

3081				CO281-31N				E318N				E32511			
Panel^§^	Phage treatment	Mean OD	Best MOIs*	Panel	Phage treatment	Mean OD	Best MOIs	Panel	Phage treatment	Mean OD	Best MOIs	Panel	Phage treatment	Mean OD	Best MOIs
A	T4	0.009	>0.1	A	T5 + T1 + T4	0.001	Any	A	T5 + T1	0.002	Any	A	T5	0.003	>0.01
	T1 + T4	0.009	>0.1		T1 + T4 + rV5	0.001	Any		T5 + T1 + T4 + rV5	0.003	Any		T5 + T1	0.004	>0.01
	T1 + T4 + rV5	0.012	>0.1		T1	0.001	Any		T5 + T1 + T4	0.004	Any		T5 + T4	0.005	>0.01
	T5 + T1 + T4 + rV5	0.013	>0.1		T1 + T4	0.001	Any		T5 + T4	0.004	>0.01		T5 + T1 + T4 + rV5	0.005	>0.01
B	T5 + T4	0.027	>0.1		T5 + T1 + T4 + rV5	0.001	Any		T5	0.004	≠1		T5 + T1 + T4	0.006	>0.01
	T4 + rV5	0.030	>0.1		T5 + T1	0.001	Any		T5 + T1 + rV5	0.005	Any		T5 + T1 + rV5	0.007	>0.01
	T5 + T4 + rV5	0.031	>0.1		T5	0.001	Any		T1 + T4 + rV5	0.007	>0.01	B	T4	0.013	>0.1
	T5 + T1 + T4	0.036	>0.1		T1 + rV5	0.001	Any		T4	0.007	>0.01		T5 + rV5	0.017	>0.1
C	T5	0.330	1,000		T5 + T1 + rV5	0.001	Any	B	T5 + T4 + rV5	0.011	>0.1		T5 + T4 + rV5	0.018	>0.1
	T5 + T1	0.347	1,000		T5 + T4	0.002	Any		T1	0.011	>10	C	T1 + T4 + rV5	0.026	>0.1
	T5 + T1 + rV5	0.377	1,000	B	T4	0.008	>0.01		T1 + rV5	0.011	NA		T4 + rV5	0.030	>0.1
D	T1	0.391	NA^y^	C	T5 + T4 + rV5	0.016	>0.1		T1 + T4	0.012	1000	D	T1 + T4	0.050	>0.1
	T1 + rV5	0.429	NA	D	T4 + rV5	0.042	>0.01	C	T5 + rV5	0.021	>0.1	E	rV5	0.170	>1
	T5 + rV5	0.464	NA		T5 + rV5	0.046	>0.1	D	T4 + rV5	0.048	>0.01	F	T1 + rV5	0.200	>10
	rV5	0.468	NA	E	rV5	0.253	NA	E	rV5	0.426	NA	G	T1	0.245	NA
**Average Mean OD**		0.198		**Average Mean OD**		0.025		**Average Mean OD**		0.038		**Average Mean OD**		0.053	

**Significance between Panels**				**Significance between Panels**				**Significance between Panels**				**Significance between Panels**			
A vs. B *P* < 0.001				A vs. B *P* < 0.001				A vs. B *P* < 0.001				A vs. B *P* < 0.05			
B vs. C *P* < 0.001				B vs. C *P* < 0.001				B vs. C *P* < 0.001				B vs. C *P* < 0.01			
C vs. D *P* < 0.001				C vs. D *P* < 0.001				C vs. D *P* < 0.001				C vs. D *P* < 0.001			
T5 + rV5 and rV5 did not work as compared to the control (*P* > 0.1)															
				D vs. E *P* < 0.001				D vs. E < 0.001				D vs. E *P* < 0.001			
												E vs. F *P* < 0.001			
												F vs. G *P* < 0.001			

**EDL933**				**H4420N**				**R508N**				**All 7 mixture**			
**Panel^§^**	**Phage treatment**	**Mean OD**	Best MOIs*	**Panel**	**Phage treatment**	**Mean OD**	**Best MOIs**	**Panel**	**Phage treatment**	**Mean OD**	**Best MOIs**	**Panel**	**Phage treatment**	**Mean OD**	**Best MOIs**

A	T5 + T1 + rV5	0.002	Any	A	T1	0.001	Any	A	T5 + T1 + T4	0.001	Any	A	T5 + T1 + rV5	0.003	Any
	T5 + T1 + T4	0.003	Any		T5 + T1 + T4	0.001	Any		T1	0.002	Any		T5 + T1	0.003	Any
	T5 + T1	0.003	>0.01		T1 + T4 + rV5	0.001	Any		T5 + T1	0.002	Any		T5 + T1 + T4 + rV5	0.004	Any
	T5 + T1 + T4 + rV5	0.003	>0.01		T5 + T1 + T4 + rV5	0.001	Any		T5 + T1 + T4 + rV5	0.002	Any		T5 + T1 + T4	0.004	Any
	T1 + rV5	0.004	>0.01		T1 + T4	0.001	Any		T5 + T1 + rV5	0.002	Any		T5	0.004	Any
B	T1	0.005	>1		T5 + T1 + rV5	0.001	Any		T1 + rV5	0.002	Any		T1 + T4	0.004	>0.01
	T1 + T4	0.005	>0.1		T5 + T1	0.001	Any		T1 + T4 + rV5	0.003	Any		T1 + T4 + rV5	0.006	Any
	T5	0.005	>1		T1 + rV5	0.001	Any		T1 + T4	0.003	Any		T5 + T4	0.008	>0.01
	T5 + T4	0.005	≠1 and 10	B	T5	0.004	>0.01	B	T5	0.067	>0.01	B	T4	0.029	1, 10
C	T1 + T4 + rV5	0.007	>0.1	C	T5 + T4	0.008	>0.01	C	T5 + T4	0.072	>0.1		T1	0.043	0.01, 0.1
	T4	0.008	>0.01	D	T5 + T4 + rV5	0.018	>0.1	D	T5 + rV5	0.136	>1		T4 + rV5	0.045	10
	T4 + rV5	0.008	>0.01	E	T5 + rV5	0.046	>0.1	E	T5 + T4 + rV5	0.172	>10		T5 + T4 + rV5	0.054	1, 100, 1000
	T5 + T4 + rV5	0.011	>0.1		T4	0.050	>0.1	F	rV5	0.267	NA		T5 + rV5	0.056	>0.1
	T5 + rV5	0.012	0.1	F	T4 + rV5	0.061	>0.1	G	T4 + rV5	0.285	NA		T1 + rV5	0.064	0.01
D	rV5	0.353	NA	G	rV5	0.293	NA	H	T4	0.336	NA	C	rV5	0.324	NA
**Average Mean OD**		0.029		**Average Mean OD**		0.033		**Average Mean OD**		0.090		**Average Mean OD**		0.043	

**Significance between Panels**				**Significance between Panels**				**Significance between Panels**				**Significance between Panels**			
A vs. B *P* < 0.01				A vs. B *P* < 0.05				A vs. B *P* < 0.001				A vs. B *P* < 0.001			
B vs. C *P* < 0.05				B vs. C *P* < 0.001				B vs. C *P* < 0.001				B vs. C *P* < 0.001			
C vs. D *P* < 0.001				C vs. D *P* < 0.001				C vs. D *P* < 0.001							
				D vs. E *P* < 0.001				D vs. E *P* < 0.001							
				E vs. F *P* < 0.001				E vs. F *P* < 0.001							
				F vs. G *P* < 0.001				F vs. G *P* < 0.001							
								G vs. H *P* < 0.001							

^§^Panels A-H were assigned to each phage treatment of which overall anti-O157 efficacy across times and MOIs statistically differed (*P* < 0.05) within each strain. * Best MOIs represent complete inhibition of bacterial growth (OD600 ≤ 0.01) at each sampling time; NA, Not applicable – bacterial growth was not completely inhibited at any MOI. Symbol >indicates all concentrations greater than that listed, symbol ≠ indicates all other MOIs were superior to that listed.

### Neutrality, Antagonism, and Facilitation by Phages

Interaction between phages varied (*P* < 0.001) with phage taxonomy, MOIs, targeted *E. coli* strains, temperatures, and times (Table 4), with phage combinations defined as neutral for >70% of incubations. For example, T5 + T1 + rV5, T5 + T1, T5 + T1 + T4 + rV5, T5 + T1 + T4, and T5 + T4 showed the same efficacy as T5 alone, in terms of lysing the seven-strain *E. coli* mixture at 22°C (Table 3). Antagonism among phages caused a reduction in cocktail efficacy and occurred most frequently (*P* < 0.001) with the combination of T5 + T4 + rV5 and T5 + rV5, MOI = 0.01, *E. coli* O157 strain 3081 and at 22°C, 22 h (Table 4). In contrast, antagonism among phages rarely occurred with combinations of T5 + T1, MOI = 1,000, against *E. coli* O157 strains CO281-31N and E318N after 10 h at 22°C. For example, odds ratios determined that the likelihood of phage antagonism occurring in T5 + T4 + rV5 and T5 + rV5 was 13 times greater (95% CI = 6.96–24.96; *P* < 0.001) than for T5 + T1. Antagonism was also seven times more likely (95% CI = 4.88–10.89, *P* < 0.001) for cocktails targeting 3081 instead of CO281-31N. Facilitation among phages was demonstrated at >22% of assessments using T1 + T4 + rV5 and T4 + T1, at MOI = 10 and 1,000, at 37°C (6 and 10 h) and when evaluating the bacterial mixture or EDL933. Significant facilitation in T1 + T4 + rV5 occurred in 52.7% cases, yielding higher effectiveness (*P* < 0.001) than T1, T4, or rV5 alone against CO281-31N, E318N, E32511, H4420N, and 7-strain *E. coli* mixture at 37°C. Furthermore, this enhanced efficacy was even greater (*P* < 0.001) than all other cocktails for the 7-strain *E. coli* mixture at 37°C.

**TABLE 4:** Effect of phage taxonomy, bacterial strains, MOIs, incubation temperatures, and times on the likelihood of phage-phage interactions effecting *E. coli* O157.

Factors	Percentage (%) of interactions						
	Neutral	Non-neutral					
		Antagonism	Facilitation	Subtotal	Odds ratio*	95% CI^§^	*P*-value
**Temperatures, Times**							
22°C, 22 h	82.6	13.8	3.6	17.4^a^	3.12	2.07-4.69	<0.001
37°C, 10 h	87.5	8.3	4.2	12.5^b^	1.93	1.26-2.96	0.002
37°C, 6 h	88.3	8.9	2.8	11.7^b^	1.77	1.15-2.72	0.009
22°C, 12 h	88.8	11.2	0	11.2^b^	1.66	1.07-2.55	0.02
37°C, 8 h	89.6	8.7	1.7	10.4^bc^	1.51	0.97-2.34	0.07
22°C, 18 h	89.5	9.7	0.8	11^bc^	1.51	0.97-2.34	0.07
22°C, 16 h	89.6	10.4	0	10.4^bc^	1.51	0.97-2.34	0.07
22°C, 20 h	90.1	8.9	1	9.9^bc^	1.4	0.9-2.18	0.1
22°C, 14 h	90.3	9.7	0	9.7^bc^	1.36	0.88-2.13	0.2
22°C, 10 h	92.4	7.6	0	7.6^c^	Referent		
**MOIs**							
0.01	71	26.8	2.2	29^a^	12.67	8.79-18.25	<0.001
0.1	87	11.6	1.4	13^b^	3.97	2.7-5.82	<0.001
100	92.3	6.5	1.2	7.7^c^	2.11	1.40-3.18	0.0004
10	93.2	5.2	1.6	6.8^c^	1.83	1.20-2.77	0.005
1	93.8	5.1	1.1	6.2^c^	1.65	1.08-2.53	0.02
1000	96	3.1	0.9	4^d^	Referent		
**Phages**							
T5 + T4 + rV5	77.7	22.3	0	22.3^a^	13.9	7.73-24.96	<0.001
T5 + rV5	79.2	20.6	0.2	21^a^	12.52	6.96-22.52	<0.001
T4 + rV5	85.2	11.9	2.9	14.8^b^	7.61	4.19-13.86	<0.001
T1 + rV5	85.4	12.3	2.3	14.6^bc^	7.47	4.1-13.6	<0.001
T1 + T4 + rV5	89	5.2	5.8	11^cd^	5.15	2.8-9.5	<0.001
T5 + T4	90.6	9.4	0	9.4^de^	4.18	2.25-7.79	<0.001
T5 + T1 + rV5	92.3	6.9	0.8	7.5^ef^	3.28	1.74-6.2	0.0002
T1 + T4	92.9	5.2	1.9	7.1^ef^	2.97	1.56-5.64	0.001
T5 + T1 + T4	93.3	6.1	0.6	7.6^ef^	2.76	1.45-5.27	0.002
T5 + T1 + T4 + rV5	94.8	5	0.2	5.2^f^	2.07	1.06-4.04	0.03
T5 + T1	97.3	2.1	0.6	2.7^g^	Referent		
**Bacteria**							
3081	77	22	1	23^a^	7.29	4.88-10.89	<0.001
7 mixture	86	9	5	14^b^	3.61	2.38-5.48	<0.001
R508N	87	13	0	13^b^	3.34	2.2-5.09	<0.001
E32511	89	10	1	11^bc^	2.6	1.68-3.99	<0.001
H4420N	91	7	2	9^cd^	1.97	1.26-3.07	0.003
EDL933	92	6	2	8^d^	1.72	1.09-2.70	0.02
E318N	93	6	1	7^de^	1.44	0.91-2.29	0.1
CO281-31N	95	5	0	5^e^	Referent		

^§^CI, confidence interval for the odds ratio. *P-value for the likelihood of non-neutral interaction as affected by different incubation temperatures, incubation times, MOIs, phages, and strains of *E. coli* O157. ^*a–g*^Within same factors, means different letters differs (*P* < 0.05).

## Discussion

To our knowledge, this is the first systematic evaluation of the efficacy of phages of diverse genera when targeting *E. coli* O157 strains incubated at different temperatures, MOIs, and times. Generally, the individual efficacy of phages at lysing *E. coli* O157 did not predict the utility of mixed cocktails in terms of neutrality, facilitation, or antagonism as compared with their individual constituents. The inclusion of multiple phages has been generally recommended as a strategy to prevent the development of phage-resistant bacterial mutants (Brüssow, 2012; Chan et al., 2013). Kudva et al. (1999) formulated a cocktail of phages KH1, KH4, and KH5 (type not characterized) that was more effective than single phages at inactivating *E. coli* O157 at 37°C in broth culture, whereas Tomat et al. (2013) demonstrated that a cocktail of two T-even myovirus reduced *E. coli* O157 on beef to a greater extent than any single phage. In this study, a combination of T1 + T4 + rV5, in particular, possessed the highest efficacy against most *E. coli* O157 strains incubated at 37°C, although this response was less pronounced at 22°C. As phage efficacy varies with the targeted strain of *E. coli*, cocktail formulation, and incubation temperature, the selection of appropriate phages for inclusion in a cocktail is crucial for successful phage therapy and biocontrol (Chan et al., 2013; Nikolich and Filippov, 2020).

Overall, superior inhibition of *E. coli* O157 occurred at 22°C after 14-18 h of incubation, highlighting the utility of phages in biocontrol of bacterial pathogens at less than optimal temperatures for bacterial growth (Kim et al., 2007; Hong et al., 2014; Liu et al., 2015; Moye et al., 2018; Tomat et al., 2018). In general, it is anticipated that phages are more efficacious in killing bacteria at optimum growth conditions of the host where the metabolic activity of cells is the highest (Weinbauer, 2004) and the development of phage resistance is less likely (Quance and Travisano, 2009; Padfield et al., 2020). However, similar to this study, siphovirus ESP1-3 and myovirus ESP 732-1 were more effective at 24°C than 37°C in reducing numbers of *Cronobacter sakazakii* in infant formula (Kim et al., 2007). This phenomenon indicates that the performance of phages under non-optimal host growth conditions may vary with types of phages and targeted bacteria. In addition, antagonism among phages at 22°C after 14-18-h incubation was generally reduced as compared with those at the same temperature after 22 h of exposure. Nevertheless, compared to 37°C, at 22°C, a longer incubation period was required to achieve similar phage efficacy, a finding in accord to that observed when phages are used to target *E. coli* O157 in food matrices (Hong et al., 2014; Liu et al., 2015).

In agreement with previous studies (Kudva et al., 1999; Tomat et al., 2013; Hudson et al., 2015; Liu et al., 2015), a higher MOI was associated with greater efficacy of phage against *E. coli* O157. Nevertheless, in this study, some individual phages or cocktails completely inhibited bacterial growth at MOIs as low as 0.01–0.1. This outcome is likely a reflection of active phage treatment where bacteria are killed by phages that are released from primary bacterial infections (Payne et al., 2000), a distinct advantage over conventional antimicrobials, as treatment efficacy can be maintained, although the dosage of phage is reduced (Loc-Carrillo and Abedon, 2011). Active phage replication has succeeded in treating infectious diseases in mice (Alemayehu et al., 2012), humans (Payne et al., 2000), and cattle (Williams Smith et al., 1987). However, as contact between phages and targeted bacteria can be tenuous *in vivo*, an MOI of at least 10 is usually recommended to ensure that 99.99% of host bacterial cells are infected by at least one phage (Abedon, 2009). Furthermore, antagonism among phages is likely more substantial at low as compared with high MOI.

As has been previously noted (Niu et al., 2009a), the sensitivity of *E. coli* O157 strains to phages differed among phage types. *E. coli* O157 subtypes PT4, PT8, PT14, PT21, PT31, and PT87 have been associated with numerous sporadic cases and outbreaks of Shiga toxin-producing *E. coli* infections worldwide (Riley et al., 1983; Woodward et al., 2002; Mora et al., 2004; Leotta et al., 2008). Compared with other *E. coli* O157 strains, phages were less effective at killing 3081 (PT43), a bovine strain that was originally isolated from a healthy calf in the United States (Dean-Nystrom et al., 1997). Combining multiple phages did not improve efficacy against 3081, as phage antagonism was most prominent with this strain. Interestingly, this strain has also been observed to be less sensitive to other antimicrobials such as tannins (Wang et al., 2009) and colicins (Nandiwada et al., 2004). *E. coli* O157 PT43 may be prevalent in feedlot cattle (Munns et al., 2016), but it has not been linked to infections in humans.

Unexpectedly, the efficacy of T1 (AHP24) alone was better at 22°C than 37°C, although a laboratory reference phage T1 (German et al., 2006) exhibited a longer latent period and lower burst size at 25°C than at 37°C (Pollard and Woodyatt, 1964). Consequently, one would expect a superior efficacy of T1 at 37°C, although another study in our laboratory found T1 (AHP24) to be more efficacious than T4 at 4°C for the control of *E. coli* O157 on beef (Liu et al., 2015). Differing from other phages, overlay plaque assays demonstrated that T1 formed halos around plaques using R508N as a host strain (Supplementary Figure 2), suggesting production of a de-polymerase with high activity at low temperatures (Pires et al., 2016). Depolymerases are used by the phages to overcome bacterial cell surface barriers early in the infection process (Pires et al., 2016; Maciejewska et al., 2018). Several *Siphoviridae* phages have been reported to produce depolymerases, degrading polysaccharide molecules that compose the capsule, lipopolysaccharides, or biofilms (Pires et al., 2016).

In this study, a combination of T4, T1, and rV5 was the cocktail that maximized facilitation effects against *E. coli* O157. Specifically, at 37°C, facilitated interactions of T4, T1, and rV5 dramatically improved efficacy against all *E. coli* O157 strains but 3081. Interaction between lytic phages remains largely undefined. However, Schmerer et al. (2014) have demonstrated synergism between phage T7 and a colanidase-producing øKMV-like virus (J8-65) against *E. coli* K12. These researchers also proposed that this synergy likely stemmed from colanidase activity produced by phage J8-65, which would increase the access of T7 to the bacterial cell surface (Schmerer et al., 2014). In addition, Chaudhry et al. (2017) have shown that a synergistic effect occurs between phage NP1 (*Nipunavirus*, *Siphoviridae*) and NP3 (*Pbunavirus*, *Myoviridae*) when removing *P. aeruginosa* biofilms from epithelial cells culture (Chaudhry et al., 2017). Presumably, an as-of-yet unidentified triple facilitation effect may occur among T4, T1, and rV5, as two-phage combinations including rV5 were more likely to produce antagonism than facilitation in this study.

Interestingly, phage antagonism was common among cocktails of T5 + T4 + rV5 and T5 + rV5, as compared with T5 alone, which demonstrated superior efficacy among the single phage treatments. Although antagonism mechanisms among lytic phages are poorly understood, mutual exclusion (where only one phage or another multiplies, not both) had occurred when bacteria were infected by phages from different families or genera (Mertens and Hausmann, 1982). Phage antagonism may occur at various stages of the life cycle, such as upon adsorption and during DNA replication. Cairns and Payne (2008) proposed mathematical modeling; combinations of phages with dissimilar life cycles would result in fast-growing phages outcompeting slow-growing phages, thereby dominating treatment responses. They also reported that the use of unrelated phages in cocktails could also result in treatment failure due to the development of multi-phage resistance. However, antagonism may also occur among phages of similar families or genera (Adams, 1959). Antagonism phenomena were observed when three *Tequatrovirus* (e.g., T4-like phages) were used to treat *E. coli*-associated diarrhea (Bourdin et al., 2014), an outcome that was attributed to the inhibition of lysis as a result of super-infection (Abedon, 2019). Likewise, James et al. (2012) reported that siphovirus LESφ2 outnumbered two co-infecting siphoviruses when norfloxacin was used to induce phages from *P. aeruginosa* strain LESB58. In addition to phage genera and combinations, in the present study, target bacterial strains, MOIs, temperatures, and times were all able to influence phage–phage interactions. For example, at 22°C, the four-phage combination was often the most efficacious treatment, irrespective of bacterial strain targeted. In contrast, at 37°C, the four-phage cocktail was effective only against strain EDL933 and was least effective against R508N. A further experimental investigation is required to identify mechanisms involved in phage antagonism and their potential impact on the efficacy of phage cocktails against targeted hosts under various environmental conditions.

Based on the results of the present study, a thorough albeit laborious screening of lytic activity of potential phage combinations against targeted host strains under environmental conditions is recommended to maximize the efficacy of phage cocktails. In the future, identification of genetic determinants derived from phages and their host resulting in facilitated or synergistic interactions would be a useful and less laborious strategy for formulating phage cocktails. In addition, determination of receptors for these O157-infecting phages and inclusion of phages with different receptors may be another means of ensuring improved biocontrol outcomes, as formulating a cocktail with phages that recognize different receptors in the host may reduce the likelihood of rapid development of antiphage mutants as well as competition for host attachment (Gordillo Altamirano and Barr, 2021).

Due to the multiple factors evaluated in the present study (MOIs, temperatures, times, host strains, and phage combinations), we were not able to monitor potential phage resistance developed over time or measure bacterial survival *via* dilution plating after phage treatment. As the rapid development of phage resistance continues to challenge the effectiveness of phage treatment (Brüssow, 2012; Chan et al., 2013), our next steps will be to verify whether the most effective cocktail in the present study can prevent the development of antiphage mutants in a large broth culture system and other biological matrices.

## Conclusion

Formulation of phage cocktails is frequently touted as the panacea to overcoming the development of antiphage bacterial mutants. However, the present study demonstrated that phage–phage interaction can be detrimental to positive biocontrol outcomes of phage cocktails. Furthermore, facilitated and antagonistic phage interactions are determined not only by biological factors such as type of phages, dosage, and targeted strains but also by environmental conditions such as temperature and duration of exposure. Therefore, to formulate phage cocktails with superior efficacy, these biological and environmental factors need to all be considered and evaluated to ensure positive facilitation or synergy among phages and optimal biocontrol.

## Data Availability Statement

The original contributions presented in the study are included in the article/Supplementary Material, further inquiries can be directed to the corresponding authors.

## Author Contributions

YN: conceived and designed the experiments. HL: performed the experiments. YN, TM, and KS: contributed reagents, materials, and analysis tools. YN, HL, HD, RM, ES, and GW: collected and analyzed the data. YN, HL, TM, and KS: wrote and revised the manuscript. All authors read and approved the final manuscript.

## Conflict of Interest

RM was employed by the company Inner Mongolia C.P. Livestock Husbandry Co., Ltd. The remaining authors declare that the research was conducted in the absence of any commercial or financial relationships that could be construed as a potential conflict of interest.

## Supplementary Material

Supplementary Figure 1 Growth curves of *E. coli* O157 H4420N at 37°C treated and not treated with phages at each MOIs.

Supplementary Figure 2 Plaque morphology of Phage T1 as a R508N host yielded by overlay plaque assay.
