# Supplementary figures and images for: Efficacy of Individual Bacteriophages Does Not Predict Efficacy of Bacteriophage Cocktails for Control of Escherichia coli O157
Source: Front Microbiol. 2021 Feb 24;12:616712. doi: 10.3389/fmicb.2021.616712 (PMC7943454; doi:10.3389/fmicb.2021.616712)

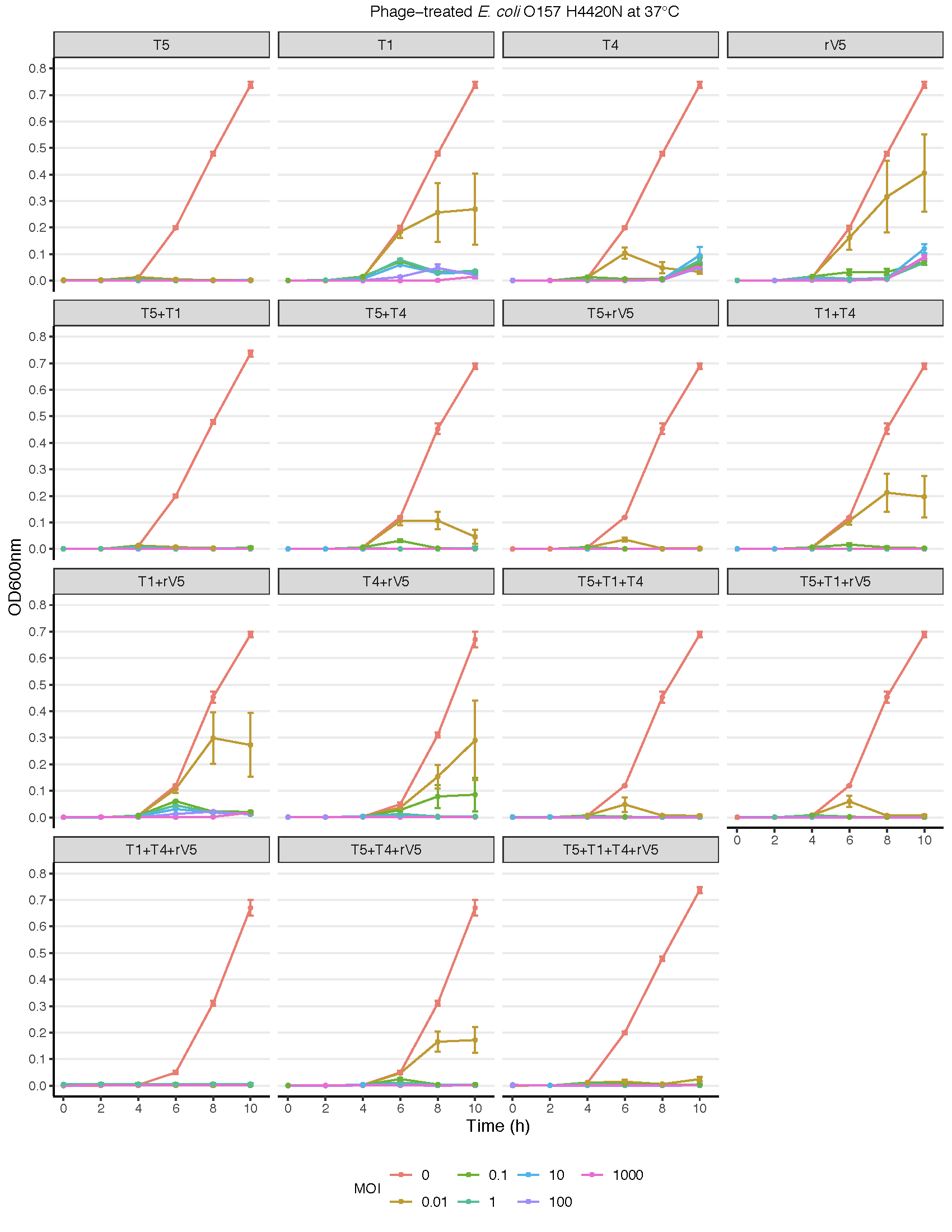

Supplement: Supplementary Figure 1 — Growth curves of E. coli O157 H4420N at 37°C treated and not treated with phages at each MOIs. [file Image_1.tiff]

**Figure S2.** Plaque morphology of Phage T1 yielded by overlay plaque assay

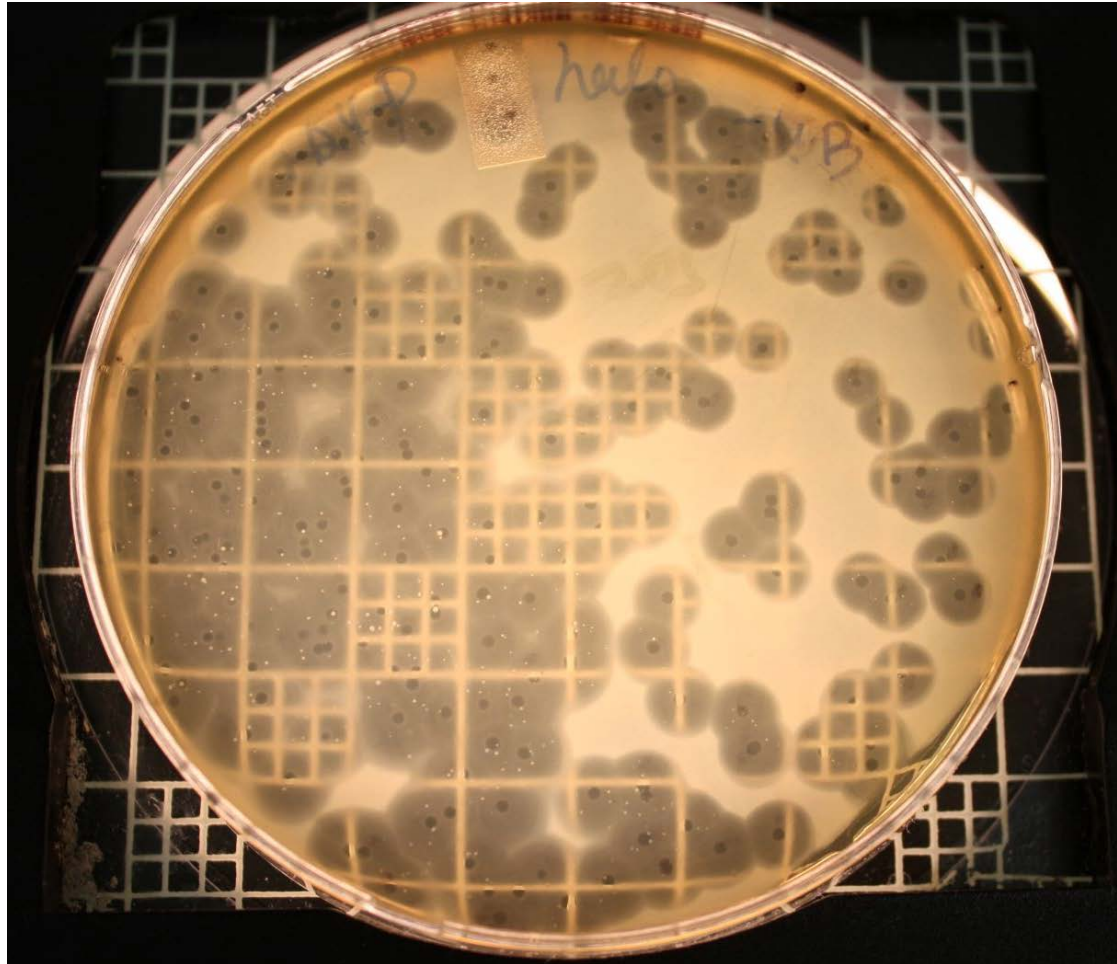

Supplement: Supplementary Figure 2 — Plaque morphology of Phage T1 as a R508N host yielded by overlay plaque assay. [file Image_2.pdf]
